# Nutritional Value, Chemical Characterization and Bulb Morphology of Greek Garlic Landraces

**DOI:** 10.3390/molecules23020319

**Published:** 2018-02-02

**Authors:** Spyridon A. Petropoulos, Ângela Fernandes, Georgia Ntatsi, Konstantinos Petrotos, Lillian Barros, Isabel C. F. R. Ferreira

**Affiliations:** 1 Department of Agriculture, University of Thessaly, Crop Production and Rural Environment, N. Ionia, 38446 Magnissia, Greece; 2 Mountain Research Centre (CIMO), ESA, Polytechnic Institute of Bragança, Campus de Santa Apolónia, 1172, 5300-253 Bragança, Portugal; afeitor@ipb.pt (Â.F.); lillian@ipb.pt (L.B.); 3 Department of Crop Production, Agricultural University of Athens, Iera Odos 75, 11855 Athens, Greece; gntatsi@aua.gr; 4 Department of Biosystems Engineering, Technological Educational Institute of Thessaly, 41110 Larissa, Greece; petrotos@teilar.gr; 5 Laboratory of Separation and Reaction Engineering-Laboratory of Catalysis and Materials (LSRE-LCM), Polytechnic Institute of Bragança, Campus de Santa Apolónia, 1172, 5300-253 Braganza, Portugal

**Keywords:** *Allium sativum* L., bulb morphology, garlic, mineral composition, pyruvic acid

## Abstract

Garlic (*Allium sativum* L.) is an important vegetable crop throughout the world. In Greece there are many areas which have specialized in garlic cultivation through the last decades, considered the main production areas. However, despite the significance of garlic as a food product and the high annual income of this crop, there is a decreasing trend in total cultivated area in Greece, and the local landraces are gradually neglected in favor of new imported genotypes. In the present study, garlic genotypes (local landraces/varieties, imported genotypes, commercial cultivars) from the main production regions of Greece were assessed for their chemical composition and quality (total soluble solids, dry matter content, nutritional value, mineral composition, organic acids, fatty acids content and free sugars content), and bulb morphology. The results of the present study showed significant diversity in quality features and bulb morphology, not only between the genotypes from different growing regions, but also between those of the same region. This result is interesting since it could be implemented for further improvement and valorization of this important vegetable crop through extensive breeding programs within the framework of sustainability and genetic, material conservation.

## 1. Introduction

Garlic (*Allium sativum* L.) is the most economically important species of the Allium genus and an important vegetable crop throughout the world [1]. In Greece, there are many areas which have specialized in garlic cultivation during the last centuries and are considered the main production areas for dry bulbs, while the crop is considered a part of the popular culture of these regions. However, despite the importance of this crop for the local communities and the high annual farmers’ income, there is a decreasing trend in total cultivated area during the last decade, whereas the local landraces that used to be the main genotypes cultivated in certain areas tend to be neglected in favor of new, more productive imported genotypes from Brazil, Chile, China, Egypt, and so on. Such a trend belies a great genetic erosion risk with many local landraces being lost during the last decades.

Garlic is considered a rich source of volatile compounds, which are responsible for the distinct flavor and the bioactive properties of dry bulbs [2]. There is also a high content of non-volatile compounds with well-known medicinal and therapeutic properties, such as amides, nitrogen oxides, phenolic compounds, especially flavonoids, proteins, saponins and sapogenins [3,4,5], as well as antioxidants, minerals (especially P, K and Se) and vitamins (especially vitamin C and vitamins of B complex) [6]. According to Koch and Lawson [7], dry garlic bulbs mainly consist of water (62–68%) and carbohydrates (26–30%), while proteins are detected in relatively less amounts (1.5–2.1%). Moreover, protein contents of 4–6% are also very common in various cultivars, considering the high dry matter content of the bulbs, while ash content ranges between 0.6% and 1.0%, and energy content is around 140 kcal 100 g^–1^ f.w. [1].

Genotype has a significant effect on the chemical composition of garlic bulbs; therefore the cultivar choice, according to the climate requirements and market quality standards, could be an important means towards the quality improvement of the final products. Despite the fact that garlic is propagated asexually with cloves in many areas of the world by farmers who use their own cloves from the previous growing season, there is also a great diversity in morphological and agronomic characters, mostly due to the existence of various ecotypes that are cultivated in the same areas for a long time and the consequent accumulation of natural mutations [8,9]. Gonzalez et al. [10] have detected significant variability in terms of organosulfur compounds, pungency, total soluble solids and antiplatelet activity, not only between garlic clones belonging in different ecophysiological groups, but also between clones of the same group. According to Mohammadi et al. [9], there was a relationship between geographical origin and genetic diversity for various Iranian garlic landraces, whereas differences in germplasm were mostly due to genotype and transfer of plant material between the various growing areas. Moreover, Singh et al. [11] analyzed the morphological variability of 47 Indian garlic collections and they suggested the existence of only two major phylogenetic groups, which could be attributed to the vegetative nature of propagation.

The existent genetic variation in garlic genotypes cultivated in Greece and its relation with the biochemical and antimicrobial properties of garlic have also been reported [12]. Fanaei et al. [13] observed significant differences in garlic pungency among various genotypes, while Jabbes et al. [14] reported significant differences in organosulfur compounds content among 31 Tunisian garlic landraces. Moreover, apart from the genotype, growing conditions and cultivation practices have also an important effect on chemical composition of garlic bulbs. In particular, fertilizer regime and soil properties may have a significant effect on bulb quality features, such as dry matter, protein and total soluble solids content, pungency and mineral composition of dry garlic bulbs [15].

Differences in bulb skin color have been associated with significant differences in chemical composition and quality of garlic genotypes. Gadel-Hak et al. [16] studied six garlic genotypes with different skin colors (white and purple skin), and they detected significant differences in vitamin C and total fractionated oil contents (higher for purple color genotypes), as well as in total phenolic compounds and flavonoids content (higher for white color genotypes). Such an interaction between bulb skin color and chemical composition was also suggested by Hong et al. [17] regarding the fructan content in garlic bulbs.

The aim of the present study was to assess various garlic genotypes, including local landraces/varieties, imported genotypes and commercial cultivars cultivated in the main growing areas of Greece, for their nutritional value, chemical composition and bulb morphological traits.

## 2. Results and Discussion

Bulb morphological features exhibited a great diversity between the studied genotypes, as presented in Table 1. Differences were observed in all the morphological features that are used to describe garlic bulbs [18], as well as in dry matter and total soluble solids content. The more profound differences were observed in average weight of cloves, where clove weight ranged from 2 (samples 3 and 14) to 15 g (sample 13), and number of cloves per bulb which ranged from 11 (sample 7) to 37 (samples 3 and 14). Fanaei et al. [13] and Mohammadi et al. [9] have also reported significant differences among Iranian garlic varieties and landraces in bulb yield, bulb weight, number of cloves per bulb and clove weight, while Jabbes et al. [14] and Singh et al. [11] have detected a great diversity in agro-morphological traits of various Tunisian and Indian garlic landraces and accessions, respectively. Dry matter and total soluble solids content ranged from as low as 31.67% (sample 9) and 31.8% (sample 10) respectively, to 42.64% (sample 11) and 40.4% (sample 12) respectively, with significant differences being observed not only between the various areas but also between genotypes of the same area. This could be attributed to different cultivation practices, soil properties and postharvest handling, as has been already reported by Diriba-Shiferaw et al. [15] and Argüello et al. [19].

However, despite the diversity between the garlics of different growing areas, similarities in most of the studied morphological features were observed between genotypes of the same growing areas, for example samples from ‘Nea Vissa’ (1 and 2), ‘Neapoli’ (3 and 14), and ‘Tripoli’ regions (11–13), indicating that adaptation to specific growing conditions and clonal selection from farmers throughout the centuries has led to uniformity of the final products [20]. Figliuolo et al. [8] suggested that selection over time according to specific agronomic traits (e.g., bulb weight and number of cloves per bulb) may be achieved by farmers, however for traits that are negatively correlated to bulb size (e.g., number of cloves per bulb and clove circumference) the adequate trade-offs have to be considered for the overall quality of the final product based on market standards [11]. Moreover, any slight differences in bulb and clove size, as well as in number of cloves per bulb, such as in the case of samples 11–13 could be attributed mainly to different cultivation practices (fertilization regime, set size, etc.), as has been already reported by Minard [21] and Rosen and Tong [22]. Since the available garlic cultivars are the result of successive accumulation of somatic mutations in ancestral mother cultivars, it is very usual for the same clone to be cultivated in different areas under different names [23].

Nutritional value of the studied garlic genotypes is presented in Table 2 and is within the range of the values suggested by Brewster [1] regarding proteins, carbohydrates, fat and energy content, and Hacıseferoğulları et al. [24] for crude protein, ash and moisture content. Significant differences were observed not only between genotypes from different regions but also between ecotypes of the same region [samples from ‘Nea Vissa’ (1 and 2), ‘Neapoli’ (3 and 14), and ‘Tripoli’ regions (11–13)], indicating that apart from the genotype, growing conditions and cultivating practices have also a significant effect on the nutritional value of garlic bulbs. Similar results have been reported by Rekowska and Skupień [6], who observed a significant decrease in dry bulb weight and diameter, and total sugar content, with increasing plant density, whereas no significant effect was observed in dry matter, reducing sugars and ash content. Moreover, fertilizer application rate and soil type has been reported to significantly affect protein content in garlic bulbs [15]. 

Mineral composition of garlic bulbs is presented in Table 3. The main minerals were K (ranging from 446 to 675 mg g^–1^ f.w.) and Ca (ranging from 163 to 963 mg g^–1^ f.w.), while Fe and Zn were also detected in considerable amounts. Apart from the differences between the genotypes from different growing areas, there were also differences between the ecotypes from the same region. According to Vadalà et al. [25] mineral composition of garlic bulbs could be used as a means for origin discrimination between samples of different growing areas in Spain, Tunisia and Italy. Hacıseferoğulları et al. [24] and Akinwande and Olatunde [26] have also reported significant amounts of K in garlic bulbs, while Mg, Na and Ca were also detected in considerable amounts. In addition, fertilizer application rates and soil properties may have a significant effect on the mineral composition of garlic bulbs, as previously supported by Diriba-Shiferaw et al. [15] Põldma et al. [27] have also reported that although Se fertilization is recommended for larger bulbs and better antioxidant activity of garlic, high Se application rates can result in the replacement of S by Se in plant metabolism and consequently the decrease in the content of essential macronutrients such as P, K and Ca.

Organic acid content of the garlic genotypes studied is presented in Table 4. The major organic acid was pyruvic acid which constituted up to 61% of total organic acids, while citric, malic and oxalic acids were detected in lower amounts. Citric and malic acid were also detected in Italian garlic varieties by Ritota et al. [28], while they additionally reported the presence of fumaric and formic acids which were not detected in the samples of the present study. This difference between the two studies regarding the organic acid composition could be due to the different assays implemented in each case (Nuclear Magnetic Resonance and Liquid Chromatography analysis). Moreover, a great diversity in organic acids composition was observed between the genotypes studied, regardless of the growing area, suggesting that apart from the genotype other factors may affect chemical composition of dry garlic bulbs. Vargas et al. [29] have reported similar results for Argentinian garlics, where they observed significant differences in the pyruvic acid content not only between different cultivars from the same area but also between the same cultivar grown in different areas. According to Põldma et al. [27] sulfur fertilizer rates may have a significant effect on pyruvic acid content and consequently on bulb pungency.

Fatty acids composition of the studied garlic genotypes is presented in Table 5. Twenty four fatty acids were detected in all the studied genotypes, with polyunsaturated fatty acids (PUFAs) having the highest content in most of the samples (41.23 to 67.56%), apart from sample 5 where saturated fatty acids (SFAs) were detected in similar amounts to PUFAs (42.91% and 41.23% respectively). The main fatty acids detected in all the studied genotypes were linoleic acid (LA, C18 : 2n6) which ranged from 32.54 to 58.97%, palmitic acid (PA, C16 : 0) which ranged from 13.62 to 18.46%, and oleic acid (C18 : 1n9) which ranged from 6.21 to 14.74%. Other fatty acids detected in lower amounts in all the studied genotypes where stearic acid (C18 : 0), α-linolenic acid (ALA, C18 : 3n3), and behenic acid (C22 : 0). Myristic acid (C14 : 0) was detected in higher amount in samples 5, 6 and 7, eicosapentaenoic acid (C20 : 5n3) in sample 4 and arachidic acid (C20 : 0) in samples 4, 5, 8, 9 and 14. Similarly, Ritota et al. [28] and Chhokar et al. [30] have also identified linoleic acid and palmitic as the main fatty acids in garlic bulbs, whereas they also reported that unsaturated fatty acids content and the ratio of unsaturated to saturated fatty acids decreases from the inner to the outer cloves of the bulb.

Sugar composition of the garlic genotypes studied is presented in Table 6. The main sugar was sucrose which ranged from 1.99 to 3.29 g 100 g^–1^ f.w. and constituted up to 97% of total sugar content. Fructose was also detected in relatively low amounts, while glucose was detected in only three of the studied genotypes (samples 1, 3 and 4). Ritota et al. [28] have also identified sucrose as the main carbohydrate in Italian garlics, while they also detected 1H signals that correspond to fructose and glucose. Moreover, Cardelle-Cobas et al. [31] studied garlic samples from Spanish markets and reported similar results to our study regarding sucrose, fructose and glucose content of dry bulbs, as well as other fructo-oligosaccharides with degree of polymerization from 3 to 7. Argüello et al. [19] have also detected significant amounts of scorodose, especially at the end of the bulbing period (about 170 days after sowing) when it was identified as the main carbohydrate.

Bhandari et al. [32] evaluated 19 garlic genotypes from South Korea, and although they reported similar amounts of sucrose (up to 3.43%) to those of our study, fructose and glucose were detected in higher amounts than our study (1.05% and 0.54% comparing to 0.41 and 0.27, for fructose and glucose respectively), which could be attributed to different genotypes and growing conditions. Significant variation according to the genotype in sugar composition and total sugar content has been previously reported by Pardo et al. [33] who evaluated 14 garlic cultivars cultivated in Spain. In most cases of that study sucrose was the main sugar followed by fructose and glucose; however there was a single case where fructose content was higher than that of sucrose (Chinese cultivar Chino Sprint). According to Rekowska and Skupień [6], sugar content can be affected by cultivation practices such as plant density, and type of ground cover. Imen et al. [34] have reported that sulfur fertilization can negatively affect reducing the carbohydrate content of rosy garlic (*Allium roseum* L.) bulbs, expressed as glucose equivalents.

Argüello et al. [19] suggested that the growth stage also has a significant effect on sugar composition, as well as vermicompost application which may result in earlier start of bulbing and consequently in higher carbohydrates content, expressed mainly by scorodose. 

## 3. Materials and Methods

### 3.1. Plant Material and Sampling

Samples of garlic bulbs were collected during the growing season of 2014–2015 (autumn to summer), at full maturity and after curing, from local farms from different regions of Greece, namely: (a) two samples of “Nea Vissa” garlic (local variety) from Evros Prefecture (samples 1 and 2); (b) three samples of “Neapoli” garlic from Laconia Prefecture, including two samples of the local landrace and one sample of Chinese origin (samples 3, 4, and 14, respectively); (c) one sample (Chinese origin) from Euboea Prefecture (sample 5); (d) two samples from Magnissia Prefecture, including one sample of “Platykampos” garlic (local landrace) and one sample of Chinese origin (samples 6 and 8); (e) three samples of “Tripoli” garlic (local variety) from Arcadia Prefecture (samples 11–13, from the regions “Stadio”, “Lithovounia” and “Mavriki”, respectively). In addition, apart from the samples collected from local farms, three more samples from garlics cultivated at the experimental farm of the University of Thessaly, Velestino, Greece, were included in the analyses, namely the local landrace “Platykampos” (sample 7), one cultivar from Messinia Prefecture (local landrace), (sample 9), and one commercial cultivar (Gardos, AGER S.A.; sample 10).

### 3.2. Standards and Reagents

Acetonitrile (99.9%), *n*-hexane (95%) and ethyl acetate (99.8%) were High Pressure Liquid Chromatography (HPLC) grade from Fisher Scientific (Lisbon, Portugal). The water was obtained from a purification system Millipore Direct-Q (TGI Pure Water Systems, Greenville, SC, USA). Fatty acids methyl esters standard mixture (standard 47885-U) sugars and organic acids standards were purchased from Sigma-Aldrich (St. Louis, MO, USA).

### 3.3. Morphological and Quality Features

**Figure 1:**
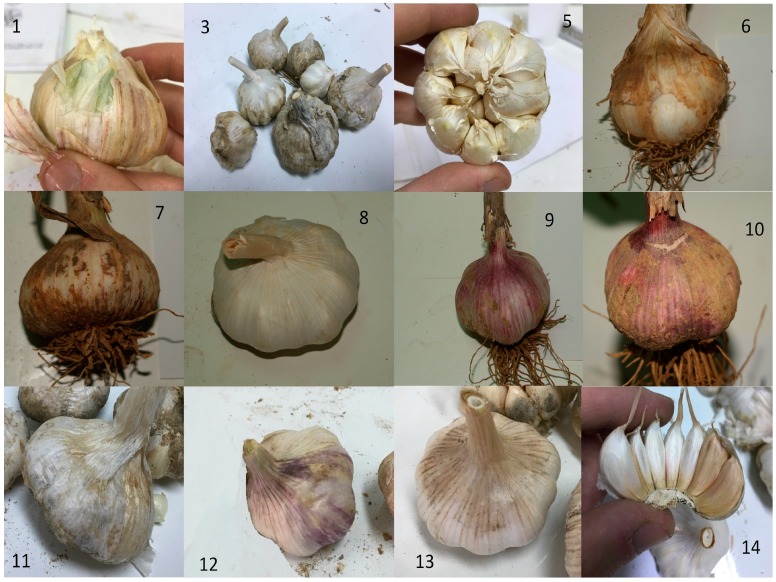
Pictures of selected genotypes and local landraces of the studied garlics. Numbers on each picture correspond to specific genotypes as described in the Materials and Methods section (Pictures with numbers 1, 3, 5, 8, 11, 12, 13, 14 have been offered by Mr. Nikolaos Polyzos).

After collection, samples from each garlic genotype were divided into three batch samples for further analyses, consisting of 15 bulbs each. Prior to chemical composition analyses, a description of morphological and quality features of bulbs for each genotype was carried out. The recorded features were the shape of bulb and bulb base, outer skin and cloves color, number of cloves, bulb structure type, bulb shape in horizontal section, the weight of 10 cloves and the dry matter content (%) and total soluble solids (°Brix) of the edible portion of cloves (Table 1). All morphology features were recorded according to International Plant Genetic Resources Institute descriptors for *Allium* spp. [18]. Pictures of selected genotypes are presented in Figure 1.

For each bulb, total soluble solids (TSS) content was measured with a hand-held refractometer (TR53000C; T.R. Turoni SRL, Forlì, Italy) on juice taken from the edible part after separating, peeling and squeezing the cloves with a kitchen garlic press. Dry matter content (%) was estimated after drying peeled cloves in a forced-air oven at 72 °C to constant weight.

### 3.4. Nutritional and Chemical Composition

For chemical composition sampling, garlic bulbs were peeled in order to obtain separate cloves, which they were further peeled and cut in slices. Samples were taken from cloves from three batches of 15 bulbs from each genotype and all the samples were stored at deep freezing conditions (−80 °C) and freeze dried prior to analysis. The freeze dried samples from each batch were powdered with pestle and mortar and stored at freezing conditions (−20 °C) until further analysis.

For macronutrients analysis, proteins, fat, carbohydrates and ash were determined using standard analytical methods described by American Organization of Analytical Chemists International (AOAC International) procedures [35]. Nitrogen content (N) was determined using the macro-Kjeldahl method, according to AOAC procedure 978.04 [36], protein content was calculated as N × 6.25. Crude fat was determined using a Soxhlet apparatus with petroleum ether, following AOAC 920.85 methodology [36]. Ash content was determined by incineration at 600 °C until a constant mass weight was achieved according to the AOAC procedures 923.03 [36]. Total carbohydrates were calculated by difference and energy was calculated following the equation: Energy (kcal) = 4 × (g protein) + 4 × (g carbohydrate) + 9 × (g fat).

For mineral composition analysis, samples of dry bulb tissue were dried in a forced-air oven at 72 °C to constant weight, ground to powder, subjected to dry ashing and extracted with 1 N HCl to determine the mineral. Ca, Mg, Fe, Mn, Zn, and Cu content were determined by atomic absorption spectrophotometry (Perkin Elmer 1100B, Waltham, MA, USA) and Na and K content by flame photometry (Sherwood Model 410, Cambridge, UK). 

Organic acids were determined following a procedure previously described by Pereira et al. [37] and the analysis was performed by ultra-fast liquid chromatography coupled to photodiode array detection (UFLC-PDA; Shimadzu Cooperation, Kyoto, Japan), using 215 nm and 245 nm (for ascorbic acid) as preferred wavelengths.

Free sugars were determined by HPLC coupled to a Refractive Index detector (Knauer, Smartline system 1000, Berlin, Germany) using the internal standard (IS, melezitose) methodology as previously described by Guimarães et al. [38] Fatty acids were determined with a gas chromatographer coupled to a flame ionization detector (GC-FID, DANI model GC 1000 instrument, Contone, Switzerland) as previously described by Guimarães et al. [38] and the results were expressed as relative percentage of each fatty acid.

### 3.5. Statistical Analysis

For nutritional and chemical composition, three samples were analyzed for each genotype, and all of the assays were carried out in triplicate. The results were expressed as mean values and standard deviation (SD). The chemical composition and antioxidant activity were analyzed using one-way analysis of variance followed by Tukey’s Honest Significant Difference (HSD) test with α = 0.05 using the Statistical Analysis Software (SAS) v. 9.1.3 statistical program (IBM Corp., Armonk, NY, USA). All the results are expressed as g/100 g f.w.

## 4. Conclusions

Overall, the Greek garlics studied showed a great diversity in their quality features and nutritional value, not only between the genotypes from different growing regions, but also between genotypes of the same region. This fact indicates that apart from genotype, growing conditions and cultivation practices may have an important effect on chemical composition and nutritional value and consequently on the quality of the final garlic products. Therefore, garlics of different regions showed similarities in terms of chemical composition and morphology, and vice-versa. Another reason that garlics of different regions were more similar than others from different regions may also be explained by transfer of plant material, especially when considering the vegetative method of propagation for the species, especially in temperate climates where this propagation method is the only one available. The existing diversity in nutritional quality is of great importance for the improvement and valorization of Greek garlics as high added value food products. Moreover, in order to discriminate and identify garlics from different regions, the results from chemical composition should be further exploited in order to create chemical fingerprinting of Greek garlics.

## Figures and Tables

**Table 1:** Morphological features of dry bulbs of the studied garlic genotypes according to International Plant Genetic Resources Institute (IPGRI, 2001) [18].

Genotypes	Bulb * Shape	Bulb Skin Colour	Clove Skin Colour	Number of Cloves	Bulb Structure Type	Shape of the Compound Bulb in Horizontal Section	Average Weight of 10 Cloves	Dry Matter (%)	TSS (Brix°)
1	3	1–6	2	4	1	1	4	37.60	37.9
2	3	1–6	2	4	1	1	4	39.91	36.4
3	2–3	1	1–2	6	1	1	1	38.86	39.4
4	3	1–6	3–5	4	1–2	1	4	35.80	35.2
5	3	1	1–2	4	1–2	1	3	32.61	33.7
6	2	2–3	2	4	1	1	4	34.02	34.2
7	3	2–3	2	4	1	1	2	32.60	34.3
8	3	1	2	5	1	1	4	35.71	37.7
9	3	1–6	2–5	4	1–2	1	2	31.67	33.6
10	3	1–6	2	4	1–2	1	3	32.27	31.8
11	2	2–3–5	3–5	4	1–2	1	4	42.64	40.4
12	2	2–3–5	3–5	6	1–2	1	4	40.52	37.6
13	2	2–3	3–5	5	1–2	1	5	36.65	36.8
14	2–3	1	1–2	6	1	1	1	33.94	39.0

* Bulb shape—1: Circular, basal plate prominent, 2: Heart-shaped, basal plate retracted, 3: Broadly ovate, basal plate even; Bulb skin colour—1 : White, 2: Cream, 3: Beige, 4: White stripes, 5: Light violet, 6: Violet, 7: Dark violet; Clove skin colour—1: White, 2: Yellow and light brown, 3: Brown, 4: Red, 5: Violet; Number of cloves-1: 1, 2: 2–4, 3: 5–10, 4: 11–15, 5: 16–20, 6: > 20, 7: Around 50; Shape of the compound bulb in horizontal section—1: Circular, 2: Elliptic; Average weight of ten cloves—1: <2 g, 2: 2–4 g, 3: >4–6 g, 4: >6–10 g, 5: >10–15 g, 6: >15 g.

**Table 2:** Nutritional value of the studied garlic genotypes (mean ± SD; *n* = 3).

Genotypes	Humidity (%)	Ash (g/100 g f.w.)	Proteins (g/100 g f.w.)	Fat (g/100 g f.w.)	Carbohydrates (g/100 g f.w.)	Energy (kcal/100 g f.w.)
1	62.38 ± 1.79 ^defg^	1.44 ± 0.02 ^fg^	6.44 ± 0.07 ^c^	0.21 ± 0.01 ^g^	29.53 ± 1.76 ^bcd^	145.76 ± 7.25 ^bcd^
2	60.09 ± 1.78 ^fgh^	1.47 ± 0.03 ^ef^	6.25 ± 0.05 ^d^	0.27 ± 0.01 ^e^	31.92 ± 1.77 ^bc^	155.08 ± 7.17 ^ab^
3	61.14 ± 0.96 ^efgh^	1.40 ± 0.03 ^g^	5.15 ± 0.07 ^g^	0.32 ± 0.01 ^bc^	31.99 ± 1.03 ^bc^	151.44 ± 3.87 ^abc^
4	64.20 ± 0.93 ^bcde^	1.65 ± 0.02 ^c^	6.23 ± 0.02 ^d^	0.30 ± 0.01 ^d^	27.62 ± 0.93 ^def^	138.16 ± 3.72 ^cdef^
5	67.39 ± 2.16 ^abc^	1.87 ± 0.01 ^a^	7.27 ± 0.05 ^b^	0.33 ± 0.02 ^b^	23.13 ± 2.19 ^h^	124.60 ± 8.72 ^f^
6	65.33 ± 1.13 ^abcd^	1.52 ± 0.03 ^de^	7.34 ± 0.05 ^ab^	0.28 ± 0.01 ^e^	25.53 ± 1.14 ^efgh^	133.96 ± 4.44 ^def^
7	67.39 ± 0.78 ^abc^	1.25 ± 0.03 ^h^	7.45 ± 0.18 ^a^	0.36 ± 0.01 ^a^	23.54 ± 0.90 ^gh^	127.20 ± 3.20 ^ef^
8	64.29 ± 1.57 ^bcde^	1.55 ± 0.04 ^d^	6.42 ± 0.02 ^c^	0.37 ± 0.01 ^a^	27.38 ± 1.58 ^defg^	138.48 ± 6.40 ^cdef^
9	68.33 ± 1.00 ^a^	1.26 ± 0.03 ^h^	6.27 ± 0.03 ^d^	0.26 ± 0.01 ^e^	23.87 ± 0.97 ^fgh^	122.94 ± 3.94 ^f^
10	67.73 ± 0.96 ^ab^	1.16 ± 0.03 ^i^	5.63 ± 0.03 ^f^	0.24 ± 0.02 ^f^	25.24 ± 0.96 ^efgh^	125.64 ± 3.85 ^ef^
11	57.36 ± 0.40 ^h^	1.87 ± 0.03 ^a^	4.62 ± 0.06 ^h^	0.12 ± 0.01 ^h^	36.03 ± 0.35 ^a^	163.69 ± 1.51 ^a^
12	59.48 ± 1.14 ^gh^	1.75 ± 0.04 ^b^	6.07 ± 0.02 ^e^	0.12 ± 0.01 ^h^	32.58 ± 1.15 ^ab^	155.69 ± 4.57 ^ab^
13	63.65 ± 1.85 ^cdef^	1.45 ± 0.03 ^efg^	6.42 ± 0.02 ^c^	0.20 ± 0.01 ^g^	28.28 ± 1.85 ^cde^	140.58 ± 7.39 ^bcde^
14	66.06 ± 0.71 ^abcd^	1.31 ± 0.02 ^h^	5.12 ± 0.04 ^g^	0.31 ± 0.01 ^cd^	27.19 ± 0.69 ^defg^	132.05 ± 2.80 ^def^

In each column different Latin letters mean significant differences between samples (*p* < 0.05).

**Table 3:** Mineral composition of the garlic genotypes studied (mg/100 g f.w.; mean values ± SD; *n* = 3).

G *	K	Na	Ca	Mg	Mn	Fe	Zn
1	529 ± 16 ^de^	18.1 ± 1.5 ^d^	359 ± 47 ^c^	23.1 ± 4.3 ^e^	1.39 ± 0.08 ^abc^	3.52 ± 0.64 ^efg^	0.81 ± 0.08 ^cde^
2	492 ± 5 ^ef^	12.0 ± 1.6 ^e^	551 ± 76 ^b^	49.0 ± 5.2 ^b^	1.39 ± 0.20 ^abc^	5.12 ± 0.32 ^abc^	0.69 ± 0.18 ^defg^
3	446 ± 47 ^g^	36.0 ± 3.5 ^a^	321 ± 78 ^cd^	33.9 ± 2.7 ^cde^	1.26 ± 0.16 ^bc^	3.45 ± 0.36 ^efg^	0.55 ± 0.06 ^g^
4	508 ± 7 ^ef^	12.4 ± 2.1 ^e^	332 ± 49 ^cd^	37.5 ± 13.0 ^bc^	1.31 ± 0.19 ^bc^	5.43 ± 0.57 ^ab^	0.86 ± 0.06 ^cd^
5	583 ± 29 ^bc^	23.9 ± 2.1 ^b^	259 ± 42 ^d^	31.7 ± 1.9 ^cde^	1.23 ± 0.20 ^c^	4.62 ± 0.81 ^bcd^	1.52 ± 0.21 ^a^
6	605 ± 48 ^b^	12.1 ± 1.4 ^e^	347 ± 73 ^cd^	32.9 ± 9.3 ^cde^	1.35 ± 0.06 ^abc^	2.89 ± 0.24 ^g^	0.82 ± 0.08 ^cde^
7	472 ± 4 ^fg^	20.0 ± 1.4 ^cd^	163 ± 30 ^e^	30.2 ± 7.0 ^cde^	1.44 ± 0.12 ^ab^	3.37 ± 0.92 ^fg^	0.83 ± 0.04 ^cde^
8	552 ± 25 ^cd^	12.1 ± 1.2 ^e^	404 ± 22 ^c^	31.4 ± 5.0 ^cde^	1.37 ± 0.07 ^abc^	4.04 ± 0.85 ^def^	1.28 ± 0.21 ^b^
9	469 ± 13 ^fg^	22.8 ± 2.9 ^bc^	361 ± 37 ^c^	25.5 ± 1.3 ^de^	1.29 ± 0.07 ^bc^	2.88 ± 0.21 ^g^	0.89 ± 0.06 ^c^
10	480 ± 21 ^fg^	19.4 ± 1.7 ^d^	322 ± 57 ^cd^	34.4 ± 8.2 ^cde^	1.30 ± 0.07 ^bc^	4.69 ± 0.55 ^bcd^	1.19 ± 0.07 ^b^
11	585 ± 13 ^bc^	12.8 ± 1.7 ^e^	264 ± 95 ^d^	26.4 ± 11.2 ^cde^	1.54 ± 0.04 ^a^	4.38 ± 0.65 ^cde^	0.63 ± 0.04 ^efg^
12	675 ± 19 ^a^	7.0 ± 0.5 ^f^	333 ± 19 ^cd^	25.4 ± 6.9 ^de^	1.52 ± 0.04 ^a^	5.78 ± 0.59 ^a^	0.76 ± 0.03 ^cdef^
13	603 ± 26 ^b^	7.8 ± 1.1 ^f^	5557 ± 22 ^b^	23.5 ± 5.5 ^e^	1.36 ± 0.13 ^abc^	3.75 ± 0.66 ^defg^	0.72 ± 0.12 ^cdefg^
14	505 ± 27 ^ef^	18.8 ± 0.4 ^d^	963 ± 38 ^a^	63.1 ± 2.0 ^a^	1.31 ± 0.06 ^bc^	3.27 ± 0.46 ^fg^	0.58 ± 0.06 ^fg^

* G: genotypes; In each column different Latin letters mean significant differences between samples (*p* < 0.05).

**Table 4:** Organic acids of the studied garlic genotypes (g/100 g f.w.; mean ± SD; *n* = 3).

Genotypes	Oxalic Acid	Malic Acid	Pyruvic Acid	Citric Acid	Total
1	0.20 ± 0.01 ^h^	0.48 ± 0.01 ^c^	1.67 ± 0.01 ^c^	0.61 ± 0.01 ^i^	2.96 ± 0.01 ^e^
2	0.14 ± 0.01 ^k^	0.36 ± 0.01 ^d^	1.09 ± 0.01 ^j^	0.61 ± 0.01 ^i^	2.20 ± 0.01 ^i^
3	0.30 ± 0.01 ^b^	0.60 ± 0.01 ^a^	1.30 ± 0.01 ^i^	1.07 ± 0.01 ^c^	3.27 ± 0.01 ^c^
4	0.23 ± 0.01 ^g^	0.44 ± 0.01 ^c^	1.55 ± 0.01 ^e^	0.85 ± 0.01 ^e^	3.07 ± 0.01 ^d^
5	0.35 ± 0.01 ^a^	0.54 ± 0.01 ^b^	1.77 ± 0.01 ^b^	1.20 ± 0.01 ^a^	3.86 ± 0.01 ^a^
6	0.29 ± 0.01 ^c^	0.36 ± 0.01 ^d^	1.41 ± 0.01 ^g^	0.68 ± 0.03 ^g^	2.74 ± 0.04 ^f^
7	0.27 ± 0.01 ^e^	0.31 ± 0.01 ^e^	1.53 ± 0.01 ^e^	0.60 ± 0.02 ^i^	2.72 ± 0.01 ^f^
8	0.28 ± 0.01 ^d^	0.32 ± 0.01 ^de^	1.91 ± 0.01 ^a^	1.15 ± 0.01 ^b^	3.66 ± 0.02 ^b^
9	0.27 ± 0.01 ^e^	0.54 ± 0.08 ^b^	1.06 ± 0.01 ^k^	0.89 ± 0.01 ^d^	2.77 ± 0.06 ^f^
10	0.24 ± 0.01 ^f^	0.19 ± 0.02 ^f^	1.33 ± 0.03 ^h^	0.84 ± 0.02 ^e^	2.60 ± 0.03 ^g^
11	0.11 ± 0.01 ^l^	0.20 ± 0.01 ^f^	1.46 ± 0.01 ^f^	0.60 ± 0.01 ^i^	2.38 ± 0.01 ^h^
12	0.18 ± 0.01 ^i^	0.10 ± 0.01 ^g^	1.07 ± 0.01 ^jk^	0.81 ± 0.01 ^f^	2.16 ± 0.01 ^i^
13	0.23 ± 0.01 ^g^	0.30 ± 0.01 ^e^	1.58 ± 0.01 ^d^	0.65 ± 0.02 ^h^	2.75 ± 0.02 ^f^
14	0.15 ± 0.01 ^j^	0.18 ± 0.01 ^f^	0.95 ± 0.01 ^l^	0.68 ± 0.01 ^g^	1.96 ± 0.01 ^j^

In each column different Latin letters mean significant differences between samples (*p* < 0.05).

**Table 5:** Composition of fatty acids (relative %) of the studied garlic genotypes (mean ± SD; *n* = 3).

	**Genotypes**						
**Fatty Acids**	**1**	**2**	**3**	**4**	**5**	**6**	**7**
C6 : 0	0.04 ± 0.01	0.04 ± 0.01	0.05 ± 0.01	0.03 ± 0.01	0.95 ± 0.06	0.75 ± 0.01	0.92 ± 0.01
C8 : 0	0.10 ± 0.01	0.10 ± 0.01	0.09 ± 0.01	0.07 ± 0.01	0.97 ± 0.07	0.73 ± 0.01	0.99 ± 0.01
C10 : 0	0.10 ± 0.01	0.10 ± 0.01	0.09 ± 0.01	0.07 ± 0.01	2.59 ± 0.02	1.87 ± 0.05	2.42 ± 0.03
C12 : 0	0.17 ± 0.01	0.17 ± 0.01	0.13 ± 0.01	0.14 ± 0.01	1.69 ± 0.02	1.09 ± 0.04	1.18 ± 0.01
C13 : 0	0.14 ± 0.01	0.13 ± 0.01	0.15 ± 0.01	0.08 ± 0.01	0.16 ± 0.01	0.16 ± 0.01	0.15 ± 0.01
C14 : 0	0.61 ± 0.03	0.55 ± 0.01	0.49 ± 0.01	0.40 ± 0.01	3.50 ± 0.05	2.35 ± 0.07	2.47 ± 0.01
C14 : 1	0.01 ± 0.01	0.01 ± 0.01	0.02 ± 0.01	0.36 ± 0.01	0.04 ± 0.01	0.03 ± 0.01	0.03 ± 0.01
C15 : 0	0.42 ± 0.01	0.45 ± 0.01	0.38 ± 0.01	0.49 ± 0.01	0.59 ± 0.01	0.47 ± 0.02	0.50 ± 0.01
C16 : 0	15.93 ± 0.28	16.43 ± 0.07	14.25 ± 0.03	13.62 ± 0.12	18.46 ± 0.21	17.61 ± 0.27	16.62 ± 0.06
C16 : 1	0.58 ± 0.04	0.63 ± 0.01	0.53 ± 0.01	0.46 ± 0.02	0.47 ± 0.02	0.67 ± 0.06	0.54 ± 0.03
C17 : 0	0.62 ± 0.01	0.71 ± 0.01	0.66 ± 0.01	0.72 ± 0.01	0.87 ± 0.01	0.67 ± 0.02	0.73 ± 0.01
C18 : 0	4.84 ± 0.01	4.35 ± 0.05	3.39 ± 0.04	2.85 ± 0.01	8.34 ± 0.05	5.87 ± 0.05	6.26 ± 0.01
C18 : 1n9	14.44 ± 0.05	12.64 ± 0.01	13.64 ± 0.04	6.75 ± 0.05	14.74 ± 0.06	11.80 ± 0.07	11.40 ± 0.01
C18 : 2n6	49.99 ± 0.16	49.48 ± 0.18	51.58 ± 0.12	52.14 ± 0.12	32.54 ± 0.10	44.93 ± 0.23	43.39 ± 0.04
C18 : 3n3	6.38 ± 0.07	7.52 ± 0.06	8.41 ± 0.01	9.14 ± 0.02	7.07 ± 0.01	6.55 ± 0.06	6.70 ± 0.01
C20 : 0	0.92 ± 0.04	1.02 ± 0.01	1.18 ± 0.04	2.56 ± 0.01	1.47 ± 0.04	0.71 ± 0.03	0.94 ± 0.07
C20 : 1	0.29 ± 0.01	0.28 ± 0.02	0.22 ± 0.01	0.37 ± 0.03	0.32 ± 0.01	0.17 ± 0.02	0.15 ± 0.01
C20 : 2	0.08 ± 0.01	0.06 ± 0.01	0.13 ± 0.01	0.13 ± 0.01	0.10 ± 0.01	0.12 ± 0.01	0.12 ± 0.01
C20 : 3	0.31 ± 0.01	0.33 ± 0.03	0.34 ± 0.01	0.39 ± 0.01	0.29 ± 0.02	0.24 ± 0.01	0.25 ± 0.02
C20 : 5n3	0.38 ± 0.03	0.48 ± 0.02	0.48 ± 0.01	3.50 ± 0.01	1.23 ± 0.01	0.33 ± 0.01	0.81 ± 0.01
C22 : 0	2.23 ± 0.04	2.68 ± 0.02	2.21 ± 0.02	3.73 ± 0.03	2.43 ± 0.13	1.70 ± 0.08	2.04 ± 0.01
C22 : 1n9	0.32 ± 0.01	0.35 ± 0.01	0.24 ± 0.01	0.32 ± 0.01	0.28 ± 0.01	0.18 ± 0.01	0.22 ± 0.01
C23 : 0	0.55 ± 0.03	0.72 ± 0.03	0.61 ± 0.04	0.78 ± 0.02	0.44 ± 0.01	0.51 ± 0.01	0.49 ± 0.02
C24 : 0	0.53 ± 0.02	0.77 ± 0.01	0.73 ± 0.01	0.91 ± 0.01	0.46 ± 0.01	0.49 ± 0.01	0.68 ± 0.06
Total SFA (% of total FA)	27.19 ± 0.21 ^g^	28.21 ± 0.16 ^e^	24.40 ± 0.08 ^k^	26.46 ± 0.14 ^h^	42.91 ± 0.01 ^a^	34.98 ± 0.32 ^c^	36.39 ± 0.02 ^b^
Total MUFA (% of total FA)	15.65 ± 0.01 ^b^	13.91 ± 0.04 ^d^	14.66 ± 0.04 ^c^	8.25 ± 0.05 ^k^	15.86 ± 0.05 ^a^	12.85 ± 0.03 ^e^	12.34 ± 0.03 ^f^
Total PUFA (% of total FA)	57.16 ± 0.20 ^h^	57.87 ± 0.20 ^g^	60.94 ± 0.12 ^f^	65.29 ± 0.10 ^d^	41.23 ± 0.05 ^k^	52.16 ± 0.29 ^i^	51.27 ± 0.06 ^j^
	**Genotypes**						
**Fatty Acids**	**8**	**9**	**10**	**11**	**12**	**13**	**14**
C6 : 0	0.14 ± 0.01	0.27 ± 0.01	0.22 ± 0.01	0.22 ± 0.01	0.09 ± 0.01	0.15 ± 0.01	0.02 ± 0.01
C8 : 0	0.16 ± 0.01	0.27 ± 0.01	0.22 ± 0.01	0.24 ± 0.01	0.08 ± 0.01	0.19 ± 0.01	0.47 ± 0.03
C10 : 0	0.36 ± 0.01	0.75 ± 0.01	0.57 ± 0.02	0.56 ± 0.02	0.21 ± 0.01	0.47 ± 0.02	0.17 ± 0.01
C12 : 0	0.48 ± 0.04	0.59 ± 0.01	0.51 ± 0.02	0.55 ± 0.01	0.40 ± 0.01	0.48 ± 0.01	0.29 ± 0.01
C13 : 0	0.03 ± 0.01	0.03 ± 0.01	0.03 ± 0.01	nd	nd	nd	0.07 ± 0.01
C14 : 0	0.79 ± 0.03	1.15 ± 0.01	0.99 ± 0.02	1.01 ± 0.01	0.74 ± 0.04	1.06 ± 0.01	0.50 ± 0.01
C14 : 1	0.09 ± 0.01	0.16 ± 0.01	0.40 ± 0.01	0.50 ± 0.01	0.41 ± 0.01	0.37 ± 0.01	0.40 ± 0.01
C15 : 0	0.42 ± 0.01	0.47 ± 0.01	0.52 ± 0.01	0.43 ± 0.01	0.39 ± 0.01	0.40 ± 0.01	0.41 ± 0.01
C16 : 0	14.66 ± 0.14	14.33 ± 0.02	15.76 ± 0.08	14.85 ± 0.01	14.52 ± 0.07	15.78 ± 0.12	13.73 ± 0.05
C16 : 1	0.61 ± 0.02	0.56 ± 0.03	0.64 ± 0.02	0.57 ± 0.04	0.60 ± 0.01	0.67 ± 0.01	0.60 ± 0.01
C17 : 0	0.69 ± 0.01	0.78 ± 0.02	0.77 ± 0.06	0.58 ± 0.01	0.61 ± 0.01	0.61 ± 0.01	0.76 ± 0.02
C18 : 0	3.31 ± 0.01	4.39 ± 0.01	4.15 ± 0.01	3.75 ± 0.05	3.42 ± 0.01	4.84 ± 0.01	2.62 ± 0.01
C18 : 1n9	8.65 ± 0.01	10.31 ± 0.01	9.10 ± 0.05	6.34 ± 0.07	6.21 ± 0.02	10.63 ± 0.02	7.01 ± 0.02
C18 : 2n6	49.37 ± 0.21	42.48 ± 0.01	50.23 ± 0.28	58.15 ± 0.26	58.97 ± 0.01	52.76 ± 0.05	54.66 ± 0.05
C18 : 3n3	10.45 ± 0.05	9.59 ± 0.01	9.65 ± 0.01	6.36 ± 0.09	6.77 ± 0.06	6.54 ± 0.01	9.96 ± 0.02
C20 : 0	2.00 ± 0.04	2.78 ± 0.01	0.97 ± 0.02	0.89 ± 0.04	1.01 ± 0.03	0.72 ± 0.02	1.38 ± 0.03
C20 : 1	0.34 ± 0.01	0.26 ± 0.01	0.19 ± 0.01	0.17 ± 0.01	0.18 ± 0.01	0.13 ± 0.01	0.16 ± 0.01
C20 : 2	0.16 ± 0.01	0.14 ± 0.01	0.19 ± 0.01	0.20 ± 0.01	0.15 ± 0.01	0.12 ± 0.01	0.180.01
C20 : 3	0.31 ± 0.01	0.28 ± 0.02	0.27 ± 0.01	0.31 ± 0.01	0.32 ± 0.01	0.24 ± 0.01	0.45 ± 0.01
C20 : 5n3	1.86 ± 0.11	4.59 ± 0.01	0.70 ± 0.01	1.11 ± 0.02	1.36 ± 0.01	1.08 ± 0.01	1.45 ± 0.14
C22 : 0	3.34 ± 0.10	3.88 ± 0.04	2.21 ± 0.02	1.95 ± 0.06	2.02 ± 0.01	1.45 ± 0.02	3.07 ± 0.01
C22 : 1n9	0.32 ± 0.01	0.28 ± 0.01	0.18 ± 0.01	0.27 ± 0.01	0.25 ± 0.01	0.31 ± 0.01	0.17 ± 0.01
C23 : 0	0.69 ± 0.01	0.66 ± 0.03	0.72 ± 0.06	0.52 ± 0.04	0.61 ± 0.05	0.49 ± 0.01	0.73 ± 0.01
C24 : 0	0.80 ± 0.06	1.01 ± 0.04	0.83 ± 0.05	0.47 ± 0.01	0.70 ± 0.01	0.52 ± 0.04	0.75 ± 0.01
Total SFA (% of total FA)	27.85 ± 0.12 ^f^	31.36 ± 0.01 ^d^	28.46 ± 0.31 ^e^	26.03 ± 0.02 ^i^	24.79 ± 0.08 ^j^	27.16 ± 0.08 ^g^	24.97 ± 0.08 ^j^
Total MUFA (% of total FA)	10.01 ± 0.03 ^j^	11.55 ± 0.01 ^h^	10.51 ± 0.03 ^i^	7.85 ± 0.12 ^l^	7.64 ± 0.01 ^m^	12.11 ± 0.03 ^g^	8.34 ± 0.02 ^k^
Total PUFA (% of total FA)	62.13 ± 0.15 ^e^	57.08 ± 0.01 ^h^	61.03 ± 0.28 ^f^	66.12 ± 0.14 ^c^	67.56 ± 0.06 ^a^	60.74 ± 0.05 ^f^	66.70 ± 0.10 ^b^

nd: not detected. In each row, different Latin letters mean significant differences between genotypes (*p* < 0.05). Caproic acid (C6 : 0); Caprylic acid (C8 : 0); Capric acid (C10 : 0); Lauric acid (C12 : 0); Tridecanoic acid (C13 : 0); Myristic acid (C14 : 0); Myristoleic acid (C14 : 1); Pentadecanoic acid (C15 : 0); Palmitic acid (C16 : 0); Palmitoleic acid (C16 : 1); Heptadecanoic acid (C17 : 0); Stearic acid (C18 : 0); Oleic acid (C18 : 1n9); Linoleic acid (C18 : 2n6c); α-Linolenic acid (C18 : 3n3); Arachidic acid (C20 : 0); Eicosenoic acid (C20 : 1); Eicosadienoic acid (C20 : 2); Eicosatrienoic acid (C20 : 3); Eicosapentaenoic acid (C20 : 5n3); Behenic acid (C22 : 0); Erucic acid (C22 : 1n9); Tricosanoic acid (C23 : 0); and Lignoceric acid (C24 : 0). SFA : total saturated fatty acids; MUFA : total monounsaturated fatty acids; PUFA : total polyunsaturated fatty acids.

**Table 6:** Free sugars of the studied garlic genotypes (g/100 g f.w.; mean ± SD; *n* = 3).

Genotypes	Fructose	Glucose	Sucrose	Total Sugars
1	0.13 ± 0.01 ^e^	0.11 ± 0.01 ^b^	2.15 ± 0.04 ^d^	2.39 ± 0.04 ^f^
2	0.10 ± 0.01 ^fg^	tr	2.39 ± 0.04 ^c^	2.49 ± 0.05 ^ef^
3	0.41 ± 0.03 ^a^	0.27 ± 0.01 ^a^	2.76 ± 0.01 ^b^	3.45 ± 0.03 ^a^
4	0.22 ± 0.01 ^c^	0.11 ± 0.01 ^b^	2.73 ± 0.01 ^b^	3.06 ± 0.01 ^b^
5	0.32 ± 0.01 ^b^	tr	2.75 ± 0.02 ^b^	3.07 ± 0.01 ^b^
6	0.12 ± 0.01 ^ef^	tr	1.99 ± 0.03 ^e^	2.11 ± 0.03 ^h^
7	0.21 ± 0.01 ^c^	tr	2.05 ± 0.01 ^de^	2.25 ± 0.01 ^g^
8	0.17 ± 0.01 ^d^	tr	3.29 ± 0.06 ^a^	3.46 ± 0.06 ^a^
9	0.20 ± 0.02 ^c^	tr	2.72 ± 0.12 ^b^	2.91 ± 0.13 ^c^
10	0.09 ± 0.01 ^g^	tr	2.67 ± 0.03 ^b^	2.76 ± 0.03 ^d^
11	0.09 ± 0.01 ^g^	tr	2.34 ± 0.06 ^c^	2.43 ± 0.07 ^ef^
12	0.08 ± 0.02 ^g^	tr	2.34 ± 0.13 ^c^	2.42 ± 0.14 ^f^
13	0.08 ± 0.01 ^g^	tr	2.09 ± 0.02 ^de^	2.17 ± 0.03 ^gh^
14	0.13 ± 0.01 ^e^	tr	2.43 ± 0.01 ^c^	2.56 ± 0.01 ^e^

tr: traces; In each column, different Latin letters mean significant differences between samples (*p* < 0.05).
